# Case Report: Diagnosis of Gaucher disease in a toddler with acute respiratory failure

**DOI:** 10.3389/fped.2025.1476541

**Published:** 2025-03-14

**Authors:** Sarah Householder, Ruchit Nagar, Nisarg Shah, Jodi Forward, Sean Bickerton, Pramod Mistry, E. Vincent S. Faustino

**Affiliations:** ^1^ Department of Pediatrics, Yale-New Haven Hospital, New Haven, CT, United States; ^2^ Department of Internal Medicine, Yale-New Haven Hospital, New Haven, CT, United States; ^3^ Yale School of Medicine, New Haven, CT, United States; ^4^ Secion of Hematology/Oncology, Department of Pediatrics, Yale-New Haven Hospital, New Haven, CT, United States; ^5^ Section of Digestive Diseases, Department of Internal Medicine, Yale School of Medicine, New Haven, CT, United States; ^6^ Section of Critical Care, Department of Pediatrics, Yale School of Medicine, New Haven, CT, United States

**Keywords:** Gaucher disease, stridor, pediatric ARDS, gene replacement therapy, case report

## Abstract

A 22-month-old male infant presented with cyanosis and stridor after a trivial fall and then developed acute respiratory distress. The respiratory status of the patient progressed rapidly to severe acute respiratory distress syndrome. Additional findings of hypersplenism prompted a comprehensive multidisciplinary approach and consideration of an inborn error of metabolism. Rapid whole genome sequence showed a compound heterozygote mutation in the *GBA1* gene involving a maternally inherited known pathogenic variant, p.L483P, and a paternally inherited novel likely pathogenic variant, p.P358l. The diagnosis of Gaucher disease was confirmed with low leukocyte acid β-glucosidase activity and the patient received recombinant macrophage-targeted enzyme replacement therapy. The patient eventually recovered, but subsequent work-up demonstrated severe bulbar dysfunction with evidence of aspiration. Two months after discharge, the patient arrived at the hospital in a condition of cardiac arrest after a suspected aspiration event associated with hemoptysis. This case illustrates a previously undescribed presentation of Gaucher disease and a new likely pathogenic genetic variant for Gaucher disease. It highlights the role of a multidisciplinary approach, including rapid whole-genome sequencing, to establish timely diagnosis and provide appropriate therapy for Gaucher disease.

## 1 Introduction

Acute hypoxemic respiratory failure is a common presentation among critically ill children. However, it is unusual for a previously asymptomatic child to present with significant stridor after a fall with subsequent severe acute respiratory distress syndrome (ARDS) and hypersplenism. The ultimate diagnosis of Gaucher disease represents a rare presentation of the disorder and nominates a previous genetic variant of uncertain significance (VUS) to be reclassified as “likely pathogenic.” This case highlights the diagnostic utility of rapid whole-genome sequencing (WGS) in the acute setting and questions whether newborn screening should include testing for Gaucher disease.

## 2 Case presentation

A 22-month-old male with a history of mild expressive language delay presented to the emergency department with stridor and concern for impending respiratory failure. The patient was born full-term via spontaneous vaginal delivery to a primigravid 22-year-old mother in a non-consanguineous relationship. He had a normal newborn screen (NBS). Routine physical examinations were unrevealing. At 16 months, he was brought to the emergency department with concerns about choking. A chest radiograph was suggestive of aspiration. He appeared well and was discharged home. At 18 months, he was referred to speech therapy for mild expressive language delay. A physical examination, including an abdominal examination, was unremarkable during these visits.

On the day of presentation, the patient was playing at home when he fell while running. According to his mother, he cried but did not lose consciousness; however, he developed worsening respiratory distress and cyanosis. When emergency medical services arrived, he was found to be dyspneic, gagging, and pale with an oxygen saturation of 85%, which improved with blow-by oxygen. He was also noted to have blood in the oropharynx after vomiting. In the emergency department, the patient had audible stridor, pallor, and cyanosis. Racemic epinephrine, albuterol, and naloxone were administered without improvement. The otolaryngology team intubated the patient and performed direct laryngoscopy and bronchoscopy and reported a patent airway up to the mainstem bronchi. Computerized tomography (CT) and subsequent magnetic resonance imaging (MRI) of the neck were unremarkable. The patient was admitted to the pediatric intensive care unit (PICU).

**Figure 1:**
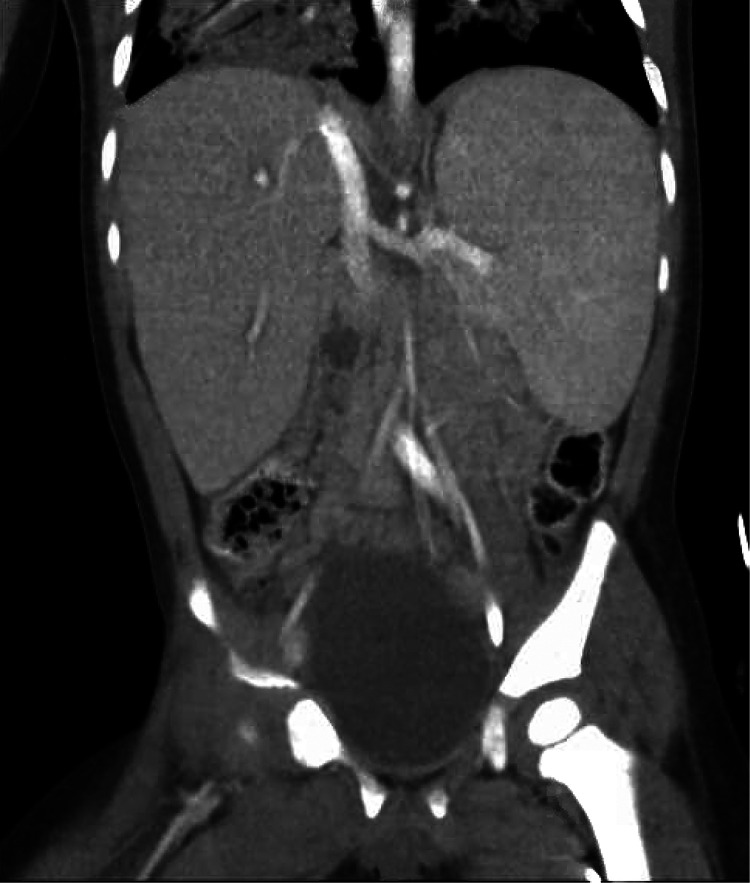
Abdominal computed tomography scan done on hospital day 2 demonstrating marked hepatosplenomegaly.

An initial physical examination in the PICU was notable for splenomegaly. His maternal aunt revealed that she also had splenomegaly without a specific diagnosis. Over the first day, the patient's hemoglobin, platelet count, and white blood cell count dropped from 12.3 to 5.8 g/dl (normal value: 10.7–113.7 g/dl), from 127,000 to 52,000/μl (normal value: 232,000–424,000/μl), and from 4,300 to 2,000/μl (normal value: 5,400–11,900/μl), respectively. A CT scan of the abdomen and pelvis showed marked hepatosplenomegaly, with the spleen measuring 11.5 cm in craniocaudal dimension, without any source of hemorrhage or signs of trauma (Figure 1). A CT scan of the chest demonstrated consolidation throughout the right-upper, right-lower, and left-lower lobe with patchy airspace opacities in the left upper lobe as well as pulmonary interstitial emphysema on the right. During the CT scan, the patient became acutely hypotensive and hypoxic, and in the context of previously low hemoglobin, he was emergently transfused unmatched packed red blood cells. His respiratory failure acutely worsened approximately 12 h afterward.

During the first few days of his admission, the patient's respiratory status progressed to severe ARDS with a peak oxygenation index of 34.9. Interventions included high frequency oscillatory ventilation with inhaled nitric oxide, paralytics, diuretics, broad-spectrum antibiotics, thoracentesis, and vasopressors. His hemoglobin levels continued to fluctuate throughout the period of admission, but he remained hemodynamically stable and did not receive additional blood products.

Given the largely unexplained severe ARDS and hypersplenism, a multidisciplinary team approach involving hematology, neurology, infectious disease, toxicology, genetics, cardiology, and pulmonology was pursued. The team considered aspiration, pneumonia, postobstructive pulmonary edema, transfusion-related acute lung injury, hemorrhagic shock, seizure, and cardiac arrhythmia as potential causes or contributors. The broad initial diagnostic work-up was unremarkable. Thus, rapid WGS and testing for inborn errors of metabolism were pursued on hospital day (HD) 3.

**Figure 2:**
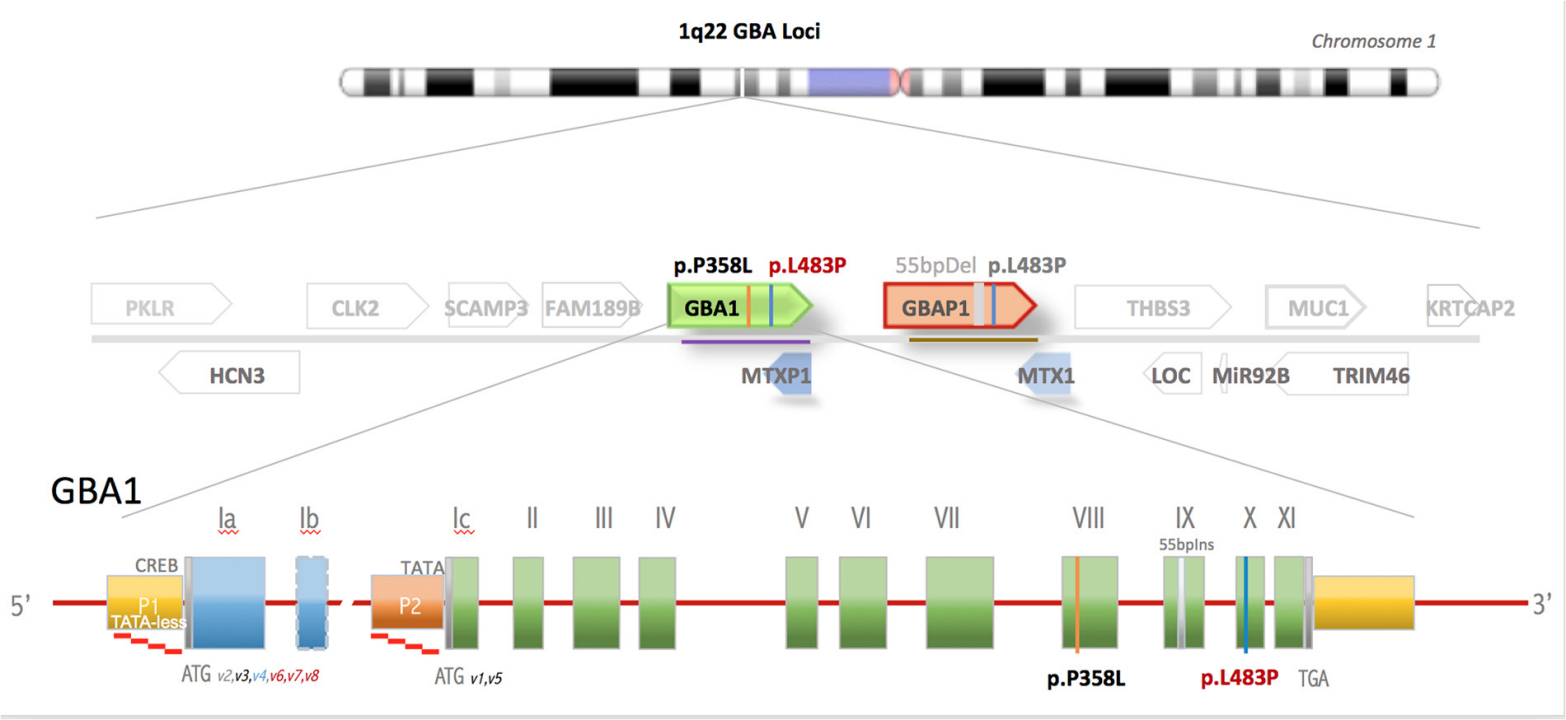
*GBA* gene locus in chromosome 1q21 is one of the most gene-dense regions of the human genome. Shown is the *GBA1* gene flanked by 15 genes, including 2 pseudogenes within 133 kB of DNA. *GBA* gene comprises 10 exons and has 2 alternative start sites. The active *GBA1* gene is closely linked to the highly homologous pseudogene, *GBAP1*, which harbors numerous mutations that, if transferred by gene conversion events to the active gene, causes Gaucher disease.

On HD8, rapid WGS resulted in two variants within the *GBA1* gene that codes for glucosylceramide β-glucosidase 1, the defective enzyme in Gaucher disease (Figure 2). One variant (p.L483P) was an established disease mutation associated with neuronal subtype of Gaucher disease when present in homozygous state. The second variant (p.P358l) was reported as VUS. Subsequent leucocyte acid β-glucosidase activity resulted as a diagnostic of Gaucher disease: 0.44 nmol/h/mg protein (normal value: >3.53 nmol/h/mg protein). Serum glucosylsphingosine, an established biomarker of Gaucher disease, was markedly elevated compared with healthy controls (patient's value: 0.199 nmol/ml, normal value: <0.004 nmol/ml). Subsequent analyses, including WGS of both parents, showed the variants in trans position, with the pathogenic variant maternally inherited and the VUS paternally inherited, fulfilling the criteria for the VUS to be nominated as “likely pathogenic.” The patient was started on enzyme replacement with imiglucerase.

The patient's respiratory status started to improve even prior to his 1st dose of imiglucerase on HD10. He was weaned from respiratory support and extubated on HD10. Despite these improvements, he showed persistent difficulty in clearing his secretions. A modified barium swallow demonstrated severe pharyngeal dysphagia with an absent swallow reflex. An MRI of the brain showed mild cortical atrophy. A gastrostomy tube was inserted before discharge to home. A month after discharge, the patient was admitted to convert his gastrostomy tube to a gastrojejunostomy tube to minimize aspiration risk. During admission, he had an aspiration event with hemoptysis after a fall, for which he received racemic epinephrine, steroids, and supplemental oxygen, and was admitted to the PICU for one day. Coagulation tests and platelet counts were within normal limits. A month after discharge, he was brought to the emergency department in a condition of cardiac arrest after another suspected aspiration event associated with hemoptysis. Sadly, the patient did not survive.

## 3 Discussion

Gaucher disease is a rare autosomal recessive lysosomal storage disorder caused by mutations in the *GBA1* gene that lead to defective lysosomal glucocerebrosidase or acid glucosidase (1). The metabolic defect leads to a cellular accumulation of glucosylceramide and glucosylsphingosine, most conspicuously seen in Gaucher cells, which are macrophages engorged with sphingolipids within their lysosomes. The multisystemic disease typically includes hepatosplenomegaly, skeletal disease, and cytopenias, and in some phenotypes neurodegenerative disease (2, 3). Gaucher disease has also been associated with spontaneous mucocutaneous bleeding, although severe bleeding is rare (4). Of the three major clinical types, the most common type (type 1) does not include neurodegenerative disease and is typically adult-onset, although it can present in childhood. Type 2 progresses rapidly, while type 3 progresses slowly. Both include neurodegenerative disease, including bulbar dysfunction, and are typically present during childhood (5).

It is unclear whether our patient had a late presentation of type 2 or a severe presentation of type 3 Gaucher disease, with bulbar dysfunction, rapidly progressive course, and early death in childhood. His visits to the emergency department and hospital admissions were likely precipitated or complicated by aspiration events related to his bulbar dysfunction. Acute pulmonary manifestations of Gaucher disease, particularly in children, are infrequently reported. Aspiration followed by pneumonitis or pneumonia and transfusion-related acute lung injury were likely contributors to the severity of our patient's ARDS. In general, in type 2 and type 3 Gaucher disease, there is pulmonary infiltrative disease due to the accumulation of Gaucher cells in lung parenchyma (2), which are capable of recruiting other immune cells to amplify the immune response and cause a cytokine storm (6). It is possible that infiltrative lung disease made our patient vulnerable to severe ARDS that was ultimately triggered by aspiration and transfusion-related acute lung injury.

A timely diagnosis of Gaucher disease is important, as treatment outcomes with enzyme replacement are generally excellent if initiated before the onset of irreversible visceral complications, although enzyme replacement has not been shown to be effective against neurologic complications. A challenge in diagnosing Gaucher disease is that the *GBA1* gene is closely linked to a highly homologous pseudogene that harbors multiple disease-causing mutations. The gene locus is vulnerable to gene conversion events that cause a transfer of pseudogene mutation to the active gene (Figure 2). Genetic panels for Gaucher disease test for about a dozen common variants, but more than 500 pathogenic variants have already been described (7). Our rapid WGS uses sequencing-by-synthesis and hybridization-based targeting and sequencing to provide quick results. However, it can miss small deletions or duplications, and it does not describe phasing, which may make the diagnosis uncertain. While standard WGS usually takes up to three months to complete, rapid WGS in our patient led to the likely diagnosis in 5 days. The diagnosis of Gaucher disease could also have been suggested biochemically, but at the time that the WGS was sent, the differential diagnosis for our patient was quite broad. WGS allowed us to screen concurrently for many diseases, including those that we had not previously considered. Furthermore, the turnaround time for biochemical markers, including leucocyte acid β-glucosidase activity, glucosylsphingosine, and chitotriosidase, is actually longer than rapid WGS in our institution. In future cases, it would be beneficial to send both rapid WGS and biochemical markers at the same time to expedite diagnosis.

The VUS gene mutation in our patient (p.P358l) is a missense mutation from a small (proline) to a large (leucine) amino acid in a protein–protein interaction site, which is predicted in computational analyses to destabilize protein structure and impair substrate binding (8). While this mutation has not been described, two other missense mutations at the same location (P358S and P358A) have been described (9, 10). Despite these predictions, functional and population studies are required to formally change the classification of the paternally inherited variant from VUS to likely pathogenic according to the recommendations of the American Genomic Society (10). Gaucher disease is currently not included in routine NBS despite the relative ease of performing the enzyme activity assay, potential consequences of delayed treatment, and availability of effective therapy. For our patient, it is possible that earlier diagnosis may have provided targeted therapies for visceral complications, home safety training for his parents if aspiration risk had been identified, and proactive management of the pulmonary manifestations of his Gaucher disease. Some states are assessing adding testing for Gaucher disease to the NBS (11, 12). We agree that it should be strongly considered for timely treatment.

In summary, this case highlights an unusual and severe presentation of neuronopathic Gaucher disease, diagnosed expeditiously through a multidisciplinary approach and rapid WGS. Moreover, WGS identified a novel, likely pathogenic, variant in an unusual presentation with ARDS, underscoring the phenotypic diversity of Gaucher disease. Finally, this case demonstrates the need for vigilance for fatal complications of Gaucher disease, that is, bulbar dysfunction, which led to the demise of our patient.

## Data Availability

The original contributions presented in the study are included in the article/Supplementary Material, and further inquiries can be directed to the corresponding author.

## References

[1] Stirnemann J Belmatoug N Camou F Serratrice C Froissart R Caillaud C A review of Gaucher disease pathophysiology, clinical presentation and treatments. Int J Mol Sci. (2017) 18(2):441. 10.3390/ijms18020441 28218669 PMC5343975

[2] Ramaswami U Mengel E Berrah A Moeenaldeen A Broomfield A Donald A Throwing a spotlight on under-recognized manifestations of Gaucher disease: pulmonary involvement, lymphadenopathy and gaucheroma. Mol Genet Metab. (2021) 133(4):335–44. 10.1016/j.ymgme.2021.06.009 34229967

[3] Lazea C Bucerzan S Al-Khzouz C Zimmermann A Cardiac manifestations in a group of Romanian patients with Gaucher disease type 1 (a monocentric study). Diagnostics. (2021) 11(6):989. 10.3390/diagnostics11060989 34072542 PMC8227770

[4] Linari S Castaman G. Hemostatic abnormalities in Gaucher disease: mechanisms and clinical implications. J Clin Med. (2022) 11(23):6920. 10.3390/jcm11236920 36498496 PMC9735904

[5] Abdelwahab M Blankenship D Schiffmann R. Long-term follow-up and sudden unexpected death in Gaucher disease type 3 in Egypt. Neurol Genet. (2016) 2(2):e55. 10.1212/NXG.0000000000000055 27123474 PMC4830203

[6] Nair S Boddupalli CS Verma R Liu J Yang R Pastores GM Type II NKT-TFH cells against Gaucher lipids regulate B-cell immunity and inflammation. Blood. (2015) 125(8):1256–71. 10.1182/blood-2014-09-600270 25499455 PMC4335081

[7] Mistry PK Lopez G Schiffmann R Barton NW Weinreb NJ Sidransky E. Gaucher disease: progress and ongoing challenges. Mol Genet Metab. (2017) 120(1–2):8–21. 10.1016/j.ymgme.2016.11.006 27916601 PMC5425955

[8] Sondka Z Dhir NB Carvalho-Silva D Jupe S Madhumita McLaren K COSMIC: a curated database of somatic variants and clinical data for cancer. Nucleic Acids Res. (2024) 52(D1):D1210–7. 10.1093/nar/gkad986 38183204 PMC10767972

[9] Buina TM Tsvetkova IV. Distribution of mutations of acid beta-D-glucosidase gene (GBA) among 68 Russian patients with Gaucher’s disease. Biomed Khim. (2007) 53(5):593–602. 18078074

[10] Chiong MAD Racoma MJC Abacan MAR. Genetic and clinical characteristics of Filipino patients with Gaucher disease. Mol Genet Metab. (2018) 15:110–5. 10.1016/j.ymgmr.2018.03.010 PMC6047105 30023299

[11] Liu J Halene S Yang M Iqbal J Yang R Mehal WZ Gaucher disease gene GBA functions in immune regulation. Proc Natl Acad Sci U S A. (2012) 109(25):10018–23. 10.1073/pnas.1200941109 22665763 PMC3382552

[12] Wasserstein MP Caggana M Bailey SM Desnick RJ Edelmann L Estrella L The New York pilot newborn screening program for lysosomal storage diseases: report of the first 65,000 infants. Genet Med. (2019) 21(3):631–40. 10.1038/s41436-018-0129-y 30093709 PMC6369014

