# Correction: Case Report: Diagnosis of Gaucher disease in a toddler with acute respiratory failure

**DOI:** 10.3389/fped.2025.1651138

**Published:** 2025-08-12

**Authors:** Sarah Householder, Ruchit Nagar, Nisarg Shah, Jodi Forward, Sean Bickerton, Pramod Mistry, E. Vincent S. Faustino

**Affiliations:** ^1^ Department of Pediatrics, Yale-New Haven Hospital, New Haven, CT, United States; ^2^ Department of Internal Medicine, Yale-New Haven Hospital, New Haven, CT, United States; ^3^ Yale School of Medicine, New Haven, CT, United States; ^4^ Secion of Hematology/Oncology, Department of Pediatrics, Yale-New Haven Hospital, New Haven, CT, United States; ^5^ Section of Digestive Diseases, Department of Internal Medicine, Yale School of Medicine, New Haven, CT, United States; ^6^ Section of Critical Care, Department of Pediatrics, Yale School of Medicine, New Haven, CT, United States

**Keywords:** Gaucher disease, stridor, pediatric ARDS, gene replacement therapy, case report

In the published article, there was an error. We refer to a specific gene mutation as “p.L484P”. However the correct gene is actually “p.L483P”.

A correction has been made to the **Abstract**. This sentence previously stated:

“Rapid whole genome sequence showed a compound heterozygote mutation in the *GBA1* gene involving a maternally inherited known pathogenic variant, p.L484P, and a paternally inherited novel likely pathogenic variant, p.P358l.”

The corrected sentence appears below:

“Rapid whole genome sequence showed a compound heterozygote mutation in the *GBA1* gene involving a maternally inherited known pathogenic variant, p.L483P, and a paternally inherited novel likely pathogenic variant, p.P358l.”

A correction has also been made to **Case Presentation**, Paragraph 6. This sentence previously stated:

“One variant (p.L484P) was an established disease mutation associated with neuronal subtype of Gaucher disease when present in homozygous state.”

The corrected sentence appears below:

“One variant (p.L483P) was an established disease mutation associated with neuronal subtype of Gaucher disease when present in homozygous state.”

The original article has been updated.

